# P-951. Delphi Study of a Fungal Curriculum for Infectious Diseases Fellows

**DOI:** 10.1093/ofid/ofae631.1141

**Published:** 2025-01-29

**Authors:** Karen Bryan, Kathleen A Linder, Marisa H Miceli, Emily Abdoler

**Affiliations:** University of Michigan, Ann Arbor, Michigan; Ann Arbor VAMC, Ann Arbor, Michigan; University of Michigan, Ann Arbor, Michigan; University of Michigan, Ann Arbor, Michigan

## Abstract

**Background:**

Infectious Diseases (ID) clinicians must be proficient in diagnosis and management of fungal diseases. Though certain fungal diseases have geographic distributions, those distributions are shifting, and ID fellows need to be prepared to practice in diverse environments. We aimed to reach consensus from experts on the critical topics to include in fungal diseases curricula for ID fellows, focusing on North and South America.

**Figure 1:**
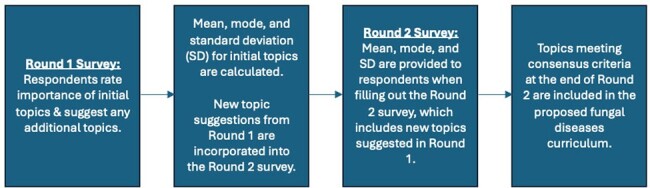
Delphi Methodology

**Methods:**

We conducted a modified Delphi survey (See Figure 1) of ID faculty in North and South America who are members of the Mycoses Study Group Education and Research Consortium (MSGERC). Authors selected the initial list of 78 topics based on a previous single institution modified Delphi, as well as fellow and expert faculty member input. Respondents rated each topic’s importance from 1 (Do Not Include) to 5 (Very Important to Include), over two survey rounds. Consensus to prioritize a topic in the curricular list was defined as ≥80% of participants rating the topic ≥ 4 (Important to Include).

**Table 1:**
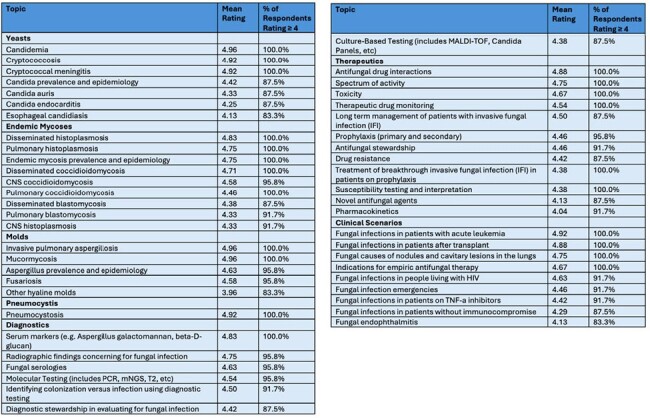
List of Topics that Met Consensus Criteria

**Results:**

Of eligible MSGERC members, 24% (33/139) responded to the first round survey and 73% of initial respondents (24/33) completed the second round. Six new topics were proposed after round 1 and were included in round 2. Fifty topics (50/84, 60%) met consensus criteria after round 2. Topics that met consensus criteria included endemic fungi and highly morbid fungal diseases, as well as specific topics regarding diagnostics and therapeutics for fungal diseases (Table 1). Some common but often less serious fungal diseases did not meet consensus, along with several rare conditions (Table 2).

**Table 2:**
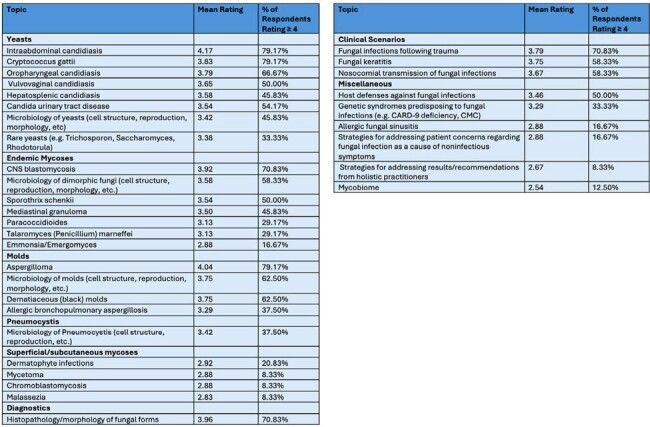
List of Topics that Did Not Meet Consensus Criteria

**Conclusion:**

The scope of fungal diseases is broad and varies geographically, which necessitates intentional prioritization of certain topics in designing curricula for ID fellows. Our survey identifies topics of particular importance in the North and South American regions. These findings, along with additional input from broad representation of ID fellowship program leaders and educational experts, can be used in the development of a standardized fungal diseases curriculum for ID fellows, regardless of the location of training.

**Disclosures:**

**Marisa H. Miceli, MD**, AN2: PI in clinical trial|F2G: PI in clinical trial|Pulmocide: PI in clinical trial|Scynexis: Advisor/Consultant|Scynexis: PI in clinical trial

